# *PEAR1* is not a major susceptibility gene for cardiovascular disease in a Flemish population

**DOI:** 10.1186/s12881-017-0411-x

**Published:** 2017-04-27

**Authors:** Wen-Yi Yang, Thibault Petit, Nicholas Cauwenberghs, Zhen-Yu Zhang, Chang-Sheng Sheng, Lutgarde Thijs, Erika Salvi, Benedetta Izzi, Christophe Vandenbriele, Fang-Fei Wei, Yu-Mei Gu, Lotte Jacobs, Lorena Citterio, Simona Delli Carpini, Cristina Barlassina, Daniele Cusi, Marc F. Hoylaerts, Peter Verhamme, Tatiana Kuznetsova, Jan A. Staessen

**Affiliations:** 1 Studies Coordinating Centre, Research Unit Hypertension and Cardiovascular Epidemiology, KU Leuven Department of Cardiovascular Sciences,, University of Leuven, Campus Sint Rafaël, Kapucijnenvoer 35, Box 7001, BE-3000 Leuven, Belgium; 2 Cardiology, Department of Cardiovascular Sciences, University of Leuven, Leuven, Belgium; 3 Genomics and Bioinformatics Platform at Filarete Foundation, Department of Health Sciences and Graduate School of Nephrology, Division of Nephrology, San Paolo Hospital, University of Milan, Milan, Italy; 4 Department of Cardiovascular Sciences, Centre for Molecular and Vascular Biology, University of Leuven, Leuven, Belgium; 5 Division of Nephrology and Dialysis, IRCCS San Raffaele Scientific Institute, University Vita-Salute San Raffaele, Milan, Italy; 6 R&D Group VitaK, Maastricht University, Maastricht, The Netherlands

**Keywords:** Clinical genetics, Cardiovascular risk, *PEAR1*, Population science, Replication research

## Abstract

**Background:**

Platelet Endothelial Aggregation Receptor 1 (PEAR1), a membrane protein highly expressed in platelets and endothelial cells, plays a role in platelet contact-induced activation, sustained platelet aggregation and endothelial function. Previous reports implicate *PEAR1 rs12041331* as a variant influencing risk in patients with coronary heart disease. We investigated whether genetic variation in *PEAR1* predicts cardiovascular outcome in a white population.

**Methods:**

In 1938 participants enrolled in the Flemish Study on Environment, Genes and Health Outcomes (51.3% women; mean age 43.6 years), we genotyped 9 tagging SNPs in *PEAR1*, measured baseline cardiovascular risk factors, and recorded Cardiovascular disease incidence. For SNPs, we contrasted cardiovascular disease incidence of minor-allele heterozygotes and homozygotes (variant) vs. major-allele homozygotes (reference) and for haplotypes carriers vs. non-carriers. In adjusted analyses, we accounted for family clusters and baseline covariables, including sex, age, body mass index, mean arterial pressure, the total-to-HDL cholesterol ratio, smoking and drinking, antihypertensive drug treatment, and history of cardiovascular disease and diabetes mellitus.

**Results:**

Over a median follow-up of 15.3 years, 238 died and 181 experienced a major cardiovascular endpoint. The multivariable-adjusted hazard ratios of eight *PEAR1* SNPs, including *rs12566888*, ranged from 0.87 to 1.07 (*P* ≥0.35) and from 0.78 to 1.30 (*P* ≥0.15), respectively. The hazard ratios of three haplotypes with frequency ≥10% ranged from 0.93 to 1.11 (*P* ≥0.49) for mortality and from 0.84 to 1.03 (*P* ≥0.29) for a cardiovascular complications. These results were not influenced by intake of antiplatelet drugs, nonsteroidal anti-inflammatory drugs, or both (*P*-values for interaction ≥ 0.056).

**Conclusions:**

In a White population, we could not replicate previous reports from experimental studies or obtained in patients suggesting that *PEAR1* might be a susceptibility gene for cardiovascular complications.

**Electronic supplementary material:**

The online version of this article (doi:10.1186/s12881-017-0411-x) contains supplementary material, which is available to authorized users.

## Background

Platelet Endothelial Aggregation Receptor 1 (PEAR1) is a membrane tyrosine kinase receptor highly expressed in platelets and endothelial cells. PEAR1 mediates platelet contact-induced activation [1] and sustains aggregation by supporting activation of the platelet specific integrin αIIbβ3 [2, 3]. Variation in the *PEAR1* gene, including SNP rs12041331, is associated with increased platelet responses to agonists [4] and with the inter-individual variability in the response to antiplatelet drugs [5, 6]. Among patients with coronary heart disease taking antiplatelet agents [5], *rs12041331 A*-allele carriers experienced more adverse cardiovascular outcomes and had higher death rates than *GG* homozygotes. Moreover, in experimental studies [7], we observed an inverse correlation between endothelial PEAR1 expression and vascular assembly. In the Heredity and Phenotype Intervention Heart Study [8], there was significant association between flow-mediated dilation and *rs12041331*. A meta-analysis of 75,000 publicly available microarrays [8] revealed that expression of PEAR1 is highly correlated with genes, such as *ANG2*, *ACVRL1* and *ENG*, and phenotypes, such as endothelial cell migration and angiogenesis, which play a pivotal role in endothelial function.

Platelet [5] and endothelial [9] dysfunction precede adverse cardiovascular outcomes, but the impact of genetic variability in *PEAR1* on cardiovascular outcome remains poorly understood and requires further clarification, in particular in unbiased population samples. To this end, we analysed the database of the Flemish Study on Environment, Genes and Health Outcomes (FLEMENGHO [10–12]) to search for association between the incidence of cardiovascular complications and genetic variation in *PEAR1*.

## Methods

### Study population

The recruitment and follow-up of FLEMENGHO participants are fully described in previous publications [10–12]. In this study, of 3343 FLEMENGHO participants, we excluded 1405 from analysis, because blood stored in the biobank was exhausted with no material left for genotyping *(n* = 521), because of DNA degradation (*n* = 314), because one or more *PEAR1* SNPs were missing (*n* = 16), because participants were less than 20 years old at enrolment (*n* = 372) with no contribution to incident cardiovascular disease, or because follow-up data were lacking (*n* = 182). Of the 16 participants with missing information on SNPs, none experienced a cardiovascular event during follow-up and one men died from prostate carcinoma. Finally, the number of participants carried through in all statistical analyses totalled 1938.

### Measurements at baseline

The information of blood pressure, anthropometric characteristics, medical history, and smoking and drinking habits were obtained by trained nurses as described elsewhere previously [12]. Blood pressure was the average of five consecutive auscultatory readings obtained with a standard mercury sphygmomanometer after participants had rested in the sitting position for at least 5 min. Mean arterial blood pressure was diastolic blood pressure plus one third of the difference of systolic minus diastolic blood pressure. The nurses also administered a standardised questionnaire inquiring about each participant’s medical history, smoking and drinking habits, and intake of medications. Antiplatelet agents included aspirin, dipyridamole, ticlopidine and clopidogrel. Plasma glucose and serum total and high-density lipoprotein (HDL) cholesterol and serum creatinine were measured by automated methods in certified laboratories.

### Follow-up of mortality and morbidity

We ascertained the vital status of participants at annual intervals until 31 December 2014 via the Belgian Population Registry. In addition, we obtained the International Classification of Disease codes for the immediate and underlying causes of death from the Flemish Registry of Death Certificates. For 1838 participants, we collected information on the incidence of non-fatal cardiovascular events either via face-to-face follow-up visits with repeated administration of the same standardised questionnaire as used at baseline (*n* = 1660) or via a structured telephone interview (*n* = 178). Follow-up data involving face-to-face contact were available from one visit in 501 participants, from two in 352, from three in 388, and from four or more in 419 participants.

Trained nurses used the International Classification of Diseases to code incident adverse health outcomes. Investigators blinded with regard to the genotypic results adjudicated the cause of death and non-fatal cardiovascular events against the medical records of general practitioners or the hospitals in the catchment area of the study. Coronary events included sudden death, fatal and non-fatal myocardial infarction, and surgical or percutaneous coronary revascularisation. Cerebrovascular disease included ischaemic stroke and transient ischaemic attack. Cardiovascular events included all of the foregoing coronary and cerebrovascular events plus pulmonary embolism, deep vein thrombosis, aortic dissection or aneurysm, and thrombosis or revascularisation of visceral or peripheral arteries. In the outcome analyses, we only considered the first event within each category.

### Genotyping

**Fig. 1:**
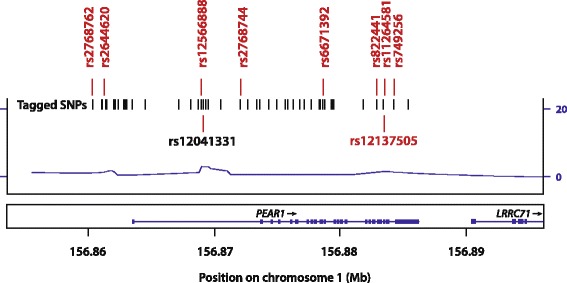
Plot of the *PEAR1* gene (p13.1–12.3). The x-axis represents the physical position on chromosome 1 (build 37, hg19). The y-axis and the line indicate the recombination rate. The nine selected tagging SNPs (rs number and position given) were in high linkage disequilibrium (*R* ^*2*^ > 0.80) with ~50 tagged SNPs denoted by vertical lines. Imputed *rs12041331* was in complete linkage with genotyped *rs12566888* (*R* ^*2*^ = 0.99; *D’* = 1.000)

*PEAR1* (22704 base-pairs) maps to a genomic area characterised by high linkage disequilibrium (*Fig.* 1) on chromosome 1. We selected nine tagging SNPs (*rs2768762*, *rs2644620*, *rs12566888*, *rs2768744*, *rs6671392*, *rs822441*, *rs11264581*, *rs12137505* and *rs749256*; Additional file 1: Table S1) that are in high linkage disequilibrium (*R* ^*2*^ > 0.80) with 53 SNPs, covering the entire *PEAR1* gene and have a minor allele frequency of at least 1%. After extraction of genomic DNA from peripheral blood cells [13], the SNPs were genotyped, using the TaqMan® OpenArray™ Genotyping System (Life Technologies, Foster City, CA). All DNA samples were loaded at 50 ng per microliter and amplified on customised arrays following the manufacturer’s instructions. For analysis of the genotypes, we used autocalling methods implemented in the TaqMan Genotyper software version 1.3 (Life Technologies). Next, genotype clusters were evaluated manually with the sample call rate set above 0.90. Sixteen duplicate samples gave 100% reproducibility for all SNPs on the custom made array [12]. The participants were genotyped for these SNPs by using the strategy as described previously [12, 13]. In our study population, *rs12041331* was in complete linkage with genotyped *rs12566888* (*R* ^*2*^ = 0.99; *D’* = 1.000). We used Minimac software [14] to impute *rs12041331*, which in previous reports [5, 15] was associated with platelet function and cardiovascular outcomes.

### Statistical analysis

For database management and statistical analysis, we used SAS software, version 9.4 (SAS Institute, Cary, NC). For comparison of means and proportions, we applied the large sample z test or ANOVA and Fisher’s exact, respectively. We tested Hardy-Weinberg equilibrium in unrelated founders, using the exact statistics available in the PROC ALLELE procedure of the SAS package. For analysis of single SNPs, we compared minor allele carriers with major allele homozygotes. We tested linkage disequilibrium, using the SAS procedures PROC ALLELE. Using the expectation-maximisation algorithm as implemented in the PROC HAPLOTYPE procedure of the SAS software, we used all SNPs included in the statistical analysis to reconstruct haplotypes. In the context of this article, haplotype refers to a set of statistically associated *PEAR1* SNPs.

We compared the cumulative incidence of adverse health outcomes in relation to genetic variants, using Cox proportional hazards models adjusted for sex and age. Next, we assessed the prognostic value of the genetic variants in multivariable-adjusted Cox regression. We checked the proportional hazard assumption by applying the Kolmogorov-type supremum test as implemented in the ASSESS statement of the PROC PHREG procedure. To account for family clusters, we used the PROC SURVIVAL procedure of the SAS-callable SUDAAN 11.0.1 software (Research Triangle Institute, NC)[12]. In this procedure, clustering within pedigrees was accounted for by including family as a random effect in the Cox models along with other covariables modelled as fixed effects. We analysed genotypes and haplotypes using major allele homozygotes and non-carriers as reference groups, respectively. Throughout our manuscript, statistical significance refers to a 2-sided *P*-value of 0.05 or less.

## Results

### Baseline characteristics

As described in a previous publication [11], all 1938 participants were White Europeans, of whom 994 (51.3%) were women. The study population consisted of 335 singletons and 1603 related subjects, belonging to 45 single-generation families and 176 multi-generation pedigrees. Age averaged (±SD) 43.6 ± 14.3 years, body mass index 25.7 ± 4.3 kg/m2, blood pressure 125.2 ± 15.7 mm Hg systolic and 76.2 ± 9.5 mm Hg diastolic, and total cholesterol 5.50 ± 1.15 mmol/L. Among all participants, 478 (24.7%) had hypertension, of whom 208 (44.0%) were on antihypertensive drug treatment, 32 (1.7%) had diabetes mellitus, and 52 (2.7%) reported a history of cardiovascular disease. Previous cardiovascular complications included coronary heart disease, ischaemic cerebrovascular disease, peripheral arterial disease and pulmonary embolism in 41 (2.1%), 9 (0.46%), 2 (0.10%) and 1 (0.052%) patients, respectively.

Of 994 women and 944 men, 275 (27.7%) women and 329 (34.8%) men were smokers, and 166 (16.7%) women and 394 (41.7%) men reported intake of alcohol. In smokers, median tobacco use was 15 cigarettes per day (interquartile range, 10 to 20 cigarettes per day). In drinkers, the median alcohol consumption was 14 g per day (8 to 28 g per day). Table 1 lists the baseline characteristics of participants according to the *rs12566888* genotype. None of the risk factors differed between minor allele carriers and major allele homozygotes (0.30 ≤ *P* ≤ 0.97).

**Table 1:** Baseline characteristics of participants by *rs12566888* genotypes

Characteristic	*T* allele carriers	*GG* homozygotes	All
N°	363	1575	1938
N° with characteristics (%)			
Women	195 (53.7)	799 (50.7)	994 (51.3)
Current smoker	101 (27.8)	503 (31.9)	604 (31.2)
Drinking alcohol	112 (30.9)	448 (28.4)	560 (28.9)
Diabetes mellitus	3 (0.8)	29 (1.8)	32 (1.7)
Hypertension	84 (23.1)	394 (25.0)	478 (24.7)
Treated hypertension	39 (10.7)	169 (10.7)	208 (10.7)
History of CVD	8 (2.2)	44 (2.8)	52 (2.7)
Antiplatelet drugs	70 (19.3)	305 (19.4)	375 (19.3)
Mean of characteristic (±SD)			
Age, years	42.8 ± 14.1	43.6 ± 14.3	43.6 ± 14.3
Body mass index, kg/m^2^	25.6 ± 4.3	25.7 ± 4.3	25.7 ± 4.3
Waist-to-hip ratio	0.84 ± 0.09	0.85 ± 0.09	0.84 ± 0.09
Systolic blood pressure, mm Hg	124.5 ± 15.2	125.3 ± 15.8	125.2 ± 15.7
Diastolic blood pressure, mm Hg	76.0 ± 9.4	76.2 ± 9.6	76.2 ± 9.5
Mean arterial pressure, mm Hg	92.2 ± 10.2	92.6 ± 10.6	92.5 ± 10.5
Heart rate, beats per minute	69.2 ± 9.5	69.4 ± 9.8	69.3 ± 9.6
Total cholesterol, mmol/L	5.47 ± 1.18	5.51 ± 1.15	5.50 ± 1.15
HDL cholesterol, mmol/L	1.39 ± 0.41	1.39 ± 0.39	1.39 ± 0.38
Total-to-HDL cholesterol ratio	4.31 ± 1.77	4.27 ± 1.65	4.27 ± 1.67
Serum creatinine, μmol/L	90.2 ± 16.6	91.0 ± 17.3	90.9 ± 17.1
Plasma glucose, mmol/L	5.12 ± 1.55	5.03 ± 1.32	5.04 ± 1.37

Abbreviations: HDL, high-density lipoprotein cholesterol. Mean arterial pressure was diastolic pressure plus one third of the difference of systolic minus diastolic pressure. Diabetes mellitus was a fasting or random plasma glucose level of ≥ 7.0 or ≥11.1 mmol/L (≥126 mg/dL or ≥200 mg/dL), or use of antidiabetic agents. Hypertension was a blood pressure of ≥140 mm Hg systolic or ≥90 mm Hg diastolic or use of antihypertensive drugs. There were no differences between minor allele carriers and major allele homozygotes (0.30 ≤ *P* ≤ 0.97)

### Incidence of events

Over a median follow-up of 15.3 years (5th to 95th percentile interval, 7.4 to 27.2 years), 238 participants died*.* Table 2 lists incident events by fatality and disease endpoint. Coronary events (*n* = 107) comprised 7 sudden deaths, 17 fatal and 35 non-fatal myocardial infarctions, 78 cases of surgical (*n* = 29) or percutaneous (*n* = 56) coronary revascularisation. Ischaemic cerebrovascular events (*n* = 61) included 15 fatal and 38 non-fatal cases of ischaemic stroke and 14 cases of transient ischaemic attack. The composite cardiovascular endpoint, consisting of 53 fatal and 128 non-fatal events, included the aforementioned coronary and cerebrovascular events plus 4 fatal and 14 non-fatal cases of pulmonary embolism or deep vein thrombosis, 7 fatal cases of aortic dissection or aneurysm, and 3 fatal and 13 non-fatal cases of thrombosis of visceral or peripheral arteries. Non-fatal events do not sum up, as only the first event in each category was analysed.

**Table 2:** Fatal and Nonfatal Cardiovascular Events

Endpoint	Type	Number of events
Sudden death	Fatal	7
Myocardial infarction	Fatal	17
	Non-fatal	35
Coronary revascularisation	Non-fatal	78
Ischaemic cardiomyopathy	Non-fatal	22
Ischaemic stroke	Fatal	15
	Non-fatal	38
Transient ischaemic attack	Non-fatal	14
Pulmonary embolism	Fatal	4
Pulmonary embolism or deep venous thrombosis	Non-fatal	14
Aortic aneurysm or dissection	Fatal	7
Peripheral arterial diseases	Fatal	2
	Non-fatal	13
Visceral arterial thrombosis	Fatal	1
Total number		181

Follow-up of the 1938 participants spanned a median of 15.3 years (5th to 95th percentile interval, 7.4–27.2 years). Participants could experience multiple non-fatal events, so that number do not add up. In the outcome analyses, only the first event within each category was considered

### Use of antiplatelet agents

Of 1938 participants, 375 (19.3%), 374 (19.3%) and 84 (4.3%) were on antiplatelet therapy, non-steroidal anti-inflammatory drugs and both at any time point, while 250 (12.9%), 240 (12.4%) and 53 (2.7%) took these agents for at least 25% of their follow-up. Of 375 patients on antiplatelet agents at any time, 369 (19.0%) took aspirin, 9 (0.46%) dipyridamole, 11 (0.57%) ticlopidine and 43 (2.2%) clopidogrel, either in monotherapy (*n* = 318 [16.4%]) or in combination (*n* = 57 [2.9%]). Among the 250 participants on antiplatelet agents during at least 25% of their follow-up, 249 (12.8%) took aspirin, 1 (0.052%) dipyridamole, 2 (0.10%) ticlopidine and 15 (7.7%) clopidogrel prescribed in monotherapy (*n* = 233 [12.0%]) or in combination (*n* = 17 [0.88%]).

### Analyses of SNPs

Additional file 1: Table S2 describes the position of the 9 SNPs on chromosome 1. In 787 unrelated founders (first generation participants), all SNPs complied with Hardy-Weinberg equilibrium (0.08 ≤ *P* ≤0.88) with the exception of *rs749256* (*P* = 0.01), which was therefore excluded for further analysis. In the whole study population (Additional file 1: Table S3), excluding *rs749256*, the frequencies of the minor alleles in all of the SNPs ranged from 9.9 to 42.9%. The frequencies of minor allele homozygotes in all of the analysed 8 SNPs were less than 5% (from 1.1 to 4.2%), except for *rs12137505* (18.5%).

**Fig. 2:**
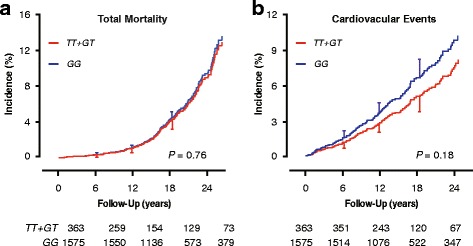
Incidence of total mortality (**a**) and cardiovascular disease (**b**) by *rs12566888* genotype. Estimates were standardised to the mean of the distributions of sex and age in the whole study population. Vertical bars denote the standard error. Cumulative incidence did not differ between minor allele homozygotes and heterozygotes (0.38 ≤ *P* ≤ 0.92). Median follow-up was 15.3 years. Tabulated data are the number of participants at risk by genotype at 6-year intervals

As illustrated for total mortality and the composite cardiovascular endpoint in relation to *rs12566888* in Fig. 2, for all SNPs (0.23 ≤ *P* ≤0.98), the sex- and age-adjusted cumulative incidence of all endpoints under study did not differ between minor allele carriers and major allele homozygotes. There were also no differences in these estimates between homozygous and heterozygous minor allele carriers (0.38 ≤ *P* ≤0.92). Next, we accounted for family clusters and adjusted the hazard ratios for baseline characteristics, including sex, age, body mass index, mean arterial pressure, the total-to-HDL cholesterol ratio, smoking and drinking, antihypertensive drug treatment and history of cardiovascular disease and diabetes mellitus. As illustrated for all endpoints under study in relation to *rs12566888*, cardiovascular risk was similar among minor allele carriers and major allele homozygotes (0.25 ≤ *P* ≤0.95; Table 3). Analysis of the seven other *PEAR1* SNPs similarly produced non-significant findings (Additional file 1: Table S4). Among the 1938 participants, 250 and 240 were taking antiplatelet agents or nonsteroidal anti-inflammatory drugs for at least 25% of their follow-up. The aforementioned results with respect to *rs12566888* and the seven other SNPs were consistent, irrespective of the intake of antiplatelet drugs, nonsteroidal anti-inflammatory drugs, or both (Additional file 1: Table S5; *P*-values for interaction ≥ 0.27).

**Table 3:** Multivariable-adjusted hazard ratios by *rs12566888* genotype

Event	N° of events (rate per 1000 person-years)		Hazard ratio (95% CI)	*P*
	*T* allele carriers (*N* = 363)	*GG* homozygotes (*N* = 1575)		
Total mortality	40 (6.80)	198 (7.58)	0.99 (0.72–1.36)	0.95
Cardiovascular mortality	7 (1.19)	46 (1.79)	0.79 (0.36–1.73)	0.55
Cardiovascular events	27 (4.76)	154 (6.15)	0.78 (0.50–1.20)	0.25
Coronary events	18 (3.13)	89 (3.50)	0.93 (0.55–1.58)	0.79
Ischaemic cerebrovascular events	8 (1.37)	53 (2.05)	0.72 (0.35–1.46)	0.36

Numbers of events do not add up, because only the first event in each category was analysed. Hazard ratios (95% confidence interval) express the risk of minor allele carriers vs. major allele homozygotes. Hazard ratios account for family clusters, and were adjusted for baseline characteristics including sex, age, body mass index, mean arterial pressure, total-to-HDL cholesterol ratio, smoking and drinking, antihypertensive drug treatment, and history of cardiovascular disease and diabetes mellitus

### Analysis of haplotypes

**Fig. 3:**
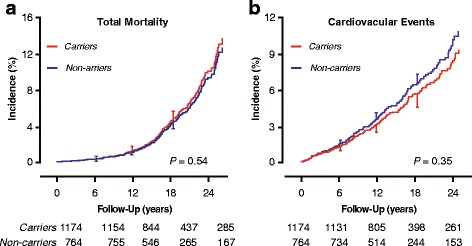
Incidence of total mortality (**a**) and cardiovascular disease (**b**) in *PEAR1 TTGATGGA* haplotype carriers and non-carriers. Letters coding the haplotype refer to the *rs2768762*, *rs2644620*, *rs12566888*, *rs2768744*, *rs6671392*, *rs822441*, *rs11264581*, and *rs12137505* alleles respectively (Additional file 1: Tables S1 and S2). Vertical bars denote the standard error. *P*-values refer to the difference between non-carriers and carriers. Median follow-up was 15.3 years. Tabulated data are the number of participants at risk by *TTGATGGA* carrying status at 6 year intervals

Using the expectation-maximisation algorithm as implemented in the PROC HAPLOTYPE procedure of the SAS software, three haplotypes had a frequency of over 10% (Additional file 1: Table S6) and were carried through in the analysis. With letters referring to the *rs2768762*, *rs2644620*, *rs12566888*, *rs2768744*, *rs6671392*, *rs822441*, *rs11264581*, and *rs12137505* alleles, respectively (Additional file 1: Tables S1 and S2), these haplotypes (Additional file 1: Table S6) were *TTGATGGA* (37.1%), *TTGATGGG* (22.8%), and *TTGATGAG* (12.0%). As illustrated for total mortality and the composite cardiovascular endpoint in relation to the most frequent haplotype (*TTGATGGA*) in Fig. 3, the sex-and age-adjusted cumulative incidence of any endpoint did not differ between carriers and non-carriers of the three haplotypes (0.24 ≤ *P* ≤0.86). Similarly, the multivariable-adjusted hazard ratios did not reveal increased risk associated with any haplotype (0.11 ≤ *P* ≤0.97; Table 4). As shown for *TTGATGGA* in Additional file 1, Table S7, the haplotype results were independent of the use of antiplatelet drugs, non-steroidal anti-inflammatory drugs, or both (*P*-values for interaction ≥ 0.056).

**Table 4:** Multivariable-adjusted hazard ratios by *PEAR1* haplotypes

Haplotypes Event	N° of events (rate per 1000 person-years)		Hazard ratio (95% CI)	*P*
	Carriers	Non carriers		
*TTGATGGA* (N° at risk)	1174	764		
Total mortality	146 (7.46)	92 (7.37)	1.05 (0.81–1.35)	0.73
Cardiovascular mortality	29 (1.48)	24 (1.92)	0.79 (0.43–1.45)	0.45
Cardiovascular events	102 (5.43)	79 (6.62)	0.84 (0.60–1.17)	0.29
Coronary events	64 (3.37)	43 (3.53)	0.98 (0.62–1.53)	0.91
Ischaemic cerebrovascular events	31 (1.60)	30 (2.43)	0.67 (0.41–1.09)	0.11
*TTGATGGG* (N° at risk)	788	1150		
Total mortality	97 (7.48)	141 (7.39)	0.93 (0.72–1.20)	0.58
Cardiovascular mortality	21 (1.62)	32 (1.68)	0.90 (0.51–1.58)	0.72
Cardiovascular events	78 (6.25)	103 (5.65)	1.03 (0.76–1.39)	0.87
Coronary events	43 (3.39)	64 (3.46)	0.93 (0.63–1.37)	0.71
Ischaemic cerebrovascular events	28 (2.18)	33 (1.75)	1.17 (0.68–2.01)	0.58
*TTGATGAG* (N° at risk)	435	1503		
Total mortality	65 (8.87)	173 (7.00)	1.11 (0.83–1.48)	0.49
Cardiovascular mortality	17 (2.32)	36 (1.46)	1.33 (0.73–2.45)	0.35
Cardiovascular events	44 (6.26)	137 (5.78)	1.00 (0.70–1.43)	0.98
Coronary events	26 (3.66)	81 (3.36)	1.01 (0.63–1.63)	0.97
Ischaemic cerebrovascular events	15 (2.07)	46 (1.88)	0.96 (0.54–1.70)	0.89

Numbers of events do not add up, because only the first event in each category was analysed. Letters coding the haplotypes refer to the *rs2768762*, *rs2644620*, *rs12566888*, *rs2768744*, *rs6671392*, *rs822441*, *rs11264581*, and *rs12137505* alleles, respectively (see Additional file 1: Tables S1 and S2). Haplotypes were reconstructed using the expectation-maximisation algorithm as implemented in the PROC HAPLOTYPE procedure of the SAS software version 9.4. Haplotypes with a frequency of ≥10% were carried forward in the analysis. Hazard ratios (95% confidence interval) express the risk of haplotype carriers vs. non carriers. Hazard ratios account for family clusters, and were adjusted for baseline characteristics including sex, age, body mass index, mean arterial pressure, total-to-HDL cholesterol ratio, smoking and drinking, antihypertensive drug treatment, and history of cardiovascular disease and diabetes mellitus

## Discussion

We could not confirm our working hypothesis that incidence of cardiovascular disease is associated with genetic variation in *PEAR1*. These results were independent of the use of antiplatelet agents or anti-inflammatory drugs. Our hypothesis originated from studies showing association of variability in the responses to antiplatelet drugs [4, 6] or of the incidence of cardiovascular complications [5] with *rs12041331*. A recent genome-wide association study [15] also demonstrated association of platelet aggregation with *rs12566888*. In our current study, we covered the entire *PEAR1* gene by analysis of eight tagging SNPs, which are in high linkage disequilibrium with 53 other SNPs in *PEAR1*. For analysis of single SNPs, we contrasted minor allele carriers with major allele homozygotes, because of the low frequency of the minor alleles and minor allele homozygotes (Additional file 1: Table S3) and because the sex- and age-adjusted cumulative incidence of all endpoints under study was similar in minor allele homozygotes and heterozygotes. *rs12566888* genotyped in the present study is a proxy for *rs12041331*. In line with the reported *R* ^2^ of 0.97 [15], in the present study population this measure of linkage disequilibrium was 0.99.

Experimental data [1, 2, 4, 15, 16] and studies of platelet aggregation in humans [17] further underpinned our hypothesis that genetic variation in *PEAR1* might predict cardiovascular risk. Indeed, recent studies [1, 2] generated the molecular and functional evidence that PEAR1 is a platelet transmembrane protein that is activated by signalling molecules or platelet contact. Agonists, such as ADP and epinephrine, enhance the membrane expression of PEAR1 and its activation by tyrosine phosphorylation, which can be blocked by eptifibatide, an αIIbβ3 antagonist [2]. Platelet proximity induced by centrifugation also increases PEAR1 tyrosine phosphorylation, independent of αIIbβ3 [2]. In a functional genomics approach, several *PEAR1* SNPs (*rs3737224*, *rs41299597*, *rs41273215*, *rs82242* and *rs11264579*) were associated with increased platelet responses to collagen-related peptide and enhanced PEAR1 protein expression [16]. Similarly, *rs12041331* [15] and *rs12566888* [4] were associated with PEAR1 expression [15] or functionality [4]. *C*-allele carriers of *rs2768759* recruited among families with premature coronary artery disease [17] showed increased native platelet aggregation to agonists in vitro before and after two weeks of aspirin treatment. Relevant for our current report, two studies [4, 15] demonstrated that platelet aggregation in response to aspirin [15] or agonists [4], including adenosine diphosphate, epinephrine and collagen, is related to intronic variation at *rs12566888*, but proposed that additional studies would be needed to clarify the importance of genetic variation in *PEAR1* to cardiovascular disease progression and response to antiplatelet therapy.

Our current null findings obtained in a general population are at variance with observations in selected patients with coronary heart disease on treatment with aspirin. Lewis and coworkers [5] addressed the association of cardiovascular outcomes with genetic variation in *PEAR1* in two independent aspirin-treated cohorts: 227 percutaneous coronary intervention patients and 1000 patients of the International Verapamil SR/Trandolapril Study Genetic Substudy (INVEST-GENES). In 144 white and 83 black patients undergoing percutaneous coronary intervention, *A*-allele carriers of *rs12041331* were more likely to experience a cardiovascular event or death compared with *GG* homozygotes. The hazard ratios were 2.62 (95% confidence interval, 0.96–7.10; *P* = 0.059) and 3.97 (1.10–14.3; *P* = 0.035), respectively). In aspirin-treated INVEST-GENES patients [5], *rs12041331 A*-allele carriers had a significantly twofold increased risk of myocardial infarction compared with *GG* homozygotes. This is in apparent contradiction with several reports showing association of the *rs12041331 A*-allele with lower platelet function or PEAR1 expression [5, 15, 18, 19] or with endothelial dysfunction [8]. This contradictory results highlight the necessity of further investigations, in particular in unbiased population samples.

The absence of association between the risk of cardiovascular complications and genetic variation in *PEAR1* demonstrates that the results from experimental studies are difficult to translate in to human health outcomes. In the Duke Databank for Cardiovascular Disease patients [20], candidate SNPs associated with in vitro aspirin resistance, including *PEAR1 rs2768759*, were not associated with clinical outcomes in aspirin-treated patients with coronary artery disease. Several issues might underlie the divergence between the experimental input and the epidemiological observations for the research question we addressed. First, the platelet function tests as used in the aforementioned genetic studies are conducted in an artificial environment, which might distort the responses to various agonists [21] and ignore the complexity of arterial thrombus formation [22]. Indeed, in clinical trials [22–24] platelet function tests set up to guide dosage of antiplatelet treatment failed to reduce cardiovascular risk. Second, as evidenced by genome-wide association studies [5], the statistical power to detect association of genetic variation with platelet function depends on other genetic variants close to the marker SNP and the frequency of the minor allele carriers, which may differ across populations and ethnicities. However, the *PEAR1* SNP frequencies in our current study are in agreement with those reported in 1000 Genomes (https://www.ncbi.nlm.nih.gov/variation/tools/1000genomes/) or HapMap (ftp://ftp.ncbi.nlm.nih.gov/hapmap/), as showed in Additional file 1: Table S8. Finally, the problem of publication bias cannot be ignored, certainly not in the field of smaller size studies with focus on a single gene. Ginsel and coworkers [25] demonstrated a biased distribution of *P*-values in abstracts listed in Medline 2012 with an apparent increase in significance levels immediately below 0.05 relative to the frequency immediately above 0.05. This finding is likely to be evidence of *P*-hacking (biased analysis and reporting) or publication bias.

## Conclusion

Based on a predefined hypothesis originating from experimental studies and observations in patients, we could not confirm that *PEAR1* is a major susceptibility gene for cardiovascular disease in the population at large. Our study illustrates that experimental studies and observations in selected patients cannot be readily extrapolated to the general population. The divergence between experimental findings and the current study result might be related to the complex network of molecular pathways, in which *PEAR1* is involved over and beyond its physiological role in platelet and endothelial function. Furthermore, epigenetic regulation of gene function might be another factor to be accounted for in future research of the functional significance of *PEAR1*.

## Additional file

Additional file 1: Table S1. Common tagging SNPs in *PEAR1.* **Table S2.** *PEAR1* allele and genotype frequencies by SNP in unrelated founders. **Table S3.** *PEAR1* allele and genotype frequencies by SNP in 1938 analysed participants. **Table S4.** Hazard ratios for all-cause mortality and the composite cardiovascular endpoint by PEAR1 SNPs. **Table S5.** Multivariable-adjusted hazard ratios associated with *rs12566888* by antiplatelet treatment status. **Table S6.** Frequencies of haplotypes. **Table S7.** Multivariable-adjusted hazard ratios associated with the *TTGATGGA* haplotype by antiplatelet treatment status. **Table S8.** Minor allele frequencies in current study population and in European subjects in 1000 Genomes and HapMap. (DOC 211 kb)

## Abbreviations

ACVRL1: Activin receptor-like kinase 1
ADP: Adenosine diphosphate
ANG2: Angiopoietin 2
CpG: -C-phosphate-G-
DNA: Deoxyribonucleic acid
ENG: Endoglin
FLEMENGHO: Flemish study on environment, genes and health outcomes
GWAS: Genome-wide association study
HDL: High-density lipoprotein
INVEST-GENES: International verapamil SR/trandolapril study genetic substudy
PEAR1: Platelet endothelial aggregation receptor 1
SNP: Single nucleotide polymorphism.
