# Supplementary material for: PEAR1 is not a major susceptibility gene for cardiovascular disease in a Flemish population
Source: BMC Med Genet. 2017 Apr 27;18:45. doi: 10.1186/s12881-017-0411-x (PMC5408434; doi:10.1186/s12881-017-0411-x)
Supplement: Additional file 1: Table S1. — Common tagging SNPs in PEAR1. Table S2. PEAR1 allele and genotype frequencies by SNP in unrelated founders. Table S3. PEAR1 allele and genotype frequencies by SNP in 1938 analysed participants. Table S4. Hazard ratios for all-cause mortality and the composite cardiovascular endpoint by PEAR1 SNPs. Table S5. Multivariable-adjusted hazard ratios associated with rs12566888 by antiplatelet treatment status. Table S6. Frequencies of haplotypes. Table S7. Multivariable-adjusted hazard ratios associated with the TTGATGGA haplotype by antiplatelet treatment status. Table S8. Minor allele frequencies in current study population and in European subjects in 1000 Genomes and HapMap. (DOC 211 kb) [file 12881_2017_411_MOESM1_ESM.doc]

**BMC Medical Genetics**

**Additional file 1**

This web appendix formed part of the original submission and has been peer reviewed.
Supplement to: *PEAR1 is not a major susceptibility gene for cardiovascular disease in the general population*.

**Table of contents**

**Table S1** Common tagging SNPs in *PEAR1* p**2**

**Table S2** *PEAR1* allele and genotype frequencies by SNP in unrelated founders p**3**

**Table S3** *PEAR1* allele and genotype frequencies by SNP in 1938 analysed participants p**4**

**Table S4** Hazard ratios for all-cause mortality and the composite cardiovascular endpoint
by PEAR1 SNPs p**5**

**Table S5** Multivariable-adjusted hazard ratios associated with *rs12566888* by antiplatelet
treatment status p**6**

**Table S6** Frequency of reconstructed haplotypes p**7**

**Table S7** Multivariable-adjusted hazard ratios associated with the *TTGATGGA* haplotype
by antiplatelet treatment status p**8**

**Table S8** Minor allele frequencies in current study population and in European subjects
in 1000 Genomes and HapMap p**9**

**Table S1**

Tagging SNPs in *PEAR1* that are in linkage disequilibrium with 53 tagged SNPs

| SNP | Position  (base pairs) | Location | Call rate | N° tagged SNPs | Tagged SNPs |
| --- | --- | --- | --- | --- | --- |
| rs2768762 | 156860462 | 5’UTR | 0.996 | 1 | rs2644619 |
| rs2644620 | 156861168 | 5’UTR | 0.998 | 24 | rs2644621, rs2644622, rs2935228, rs2986716, rs2644623, rs56146253, rs4661069, rs4661070, rs4661232, rs4233514, rs4589099, rs4661234, rs4233515, rs3118644, rs3118645, rs2935227, rs4661072, rs998459, rs72698679, rs6685635, rs2182760, rs4147300, rs4147301, rs12407092 |
| rs12566888 | 156869047 | intron | 0.998 | 6 | rs56303495, rs56106044, rs12086222, **rs12041331**, rs943549, rs6671171 |
| rs2768744 | 156872149 | intron | 0.999 | 17 | rs2768745, rs145662369, rs11264579, rs12407843, rs41267433, rs41267435, rs6688345, rs6688349, rs6664765, rs6664770, rs735953, rs12401524, rs6680819, rs77235035, rs147639000, rs11800463, rs11264580 |
| rs6671392 | 156878737 | exon | 0.999 | 5 | rs11810027, rs72698693, rs41273205, rs3737224, rs41273215 |
| rs822441 | 156882996 | exon | 0.999 | 0 |  |
| rs11264581 | 156883493 | exon | 0.999 | 0 |  |
| rs12137505 | 156883546 | exon | 0.997 | 0 |  |
| rs749256 | 156884365 | intron | 0.999 | 0 |  |

SNP ID is a GenBank ID number (National Center for Biotechnology Information, Bethesda, MD). Position and location were taken from the most recent human genome sequence assemblies (NCBI Build 37.3). Tagged SNPs have *r2* ≥ 0.80 with the *PEAR1* markers. Highlighted SNP rs12041331 was imputed and was in complete linkage disequilibrium with genotyped SNP rs12566888 (*R*^2^ = 0.99; D’ = 1.000).

**Table S2**

Allele and genotype frequencies in 787 unrelated founders

| Single nucleotides polymorphism | | | | |  | Allele and genotype frequency | | | | | | | |
| --- | --- | --- | --- | --- | --- | --- | --- | --- | --- | --- | --- | --- | --- |
| SNP | Position  (base pairs) | Base pairs | Location | *r^2^* |  | *A1* (%) | *A2* (%) |  | *A1A1* (%) | *A2A1* (%) | *A2A2* (%) |  | *P* |
| rs2768762 | 156860462 | -8585 | 5’UTR | 0.25 |  | *G* (21.5) | *T* (78.5) |  | *GG* (4.7) | *TG* (33.5) | *TT* (61.8) |  | 0.88 |
| rs2644620 | 156861168 | -7879 | 5’UTR | 0.01 |  | *C* (12.5) | *T* (87.5) |  | *TT* (1.8) | *CT* (21.5) | *CC* (76.7) |  | 0.59 |
| rs12566888 | 156869047 | 0 | intron | 0 |  | *T* (10.4) | *G* (89.6) |  | *TT* (1.7) | *GT* (17.4) | *GG* (80.9) |  | 0.08 |
| rs2768744 | 156872149 | 3102 | intron | 0.33 |  | *G* (22.0) | *A* (78.0) |  | *GG* (5.2) | *AG* (33.5) | *AA* (61.3) |  | 0.54 |
| rs6671392 | 156878737 | 9690 | exon | 0.01 |  | *C* (14.0) | *T* (86.0) |  | *CC* (2.7) | *TC* (22.6) | *TT* (74.7) |  | 0.10 |
| rs822441 | 156882996 | 13949 | exon | 0.03 |  | *C* (16.1) | *G* (83.9) |  | *CC* (3.4) | *GC* (25.3) | *GG* (71.3) |  | 0.08 |
| rs11264581 | 156883493 | 14446 | exon | 0.04 |  | *A* (16.6) | *G* (83.4) |  | *AA* (2.5) | *GA* (28.2) | *GG* (69.3) |  | 0.64 |
| rs12137505 | 156883546 | 14499 | exon | 0.005 |  | *G* (42.2) | *A* (57.8) |  | *GG* (18.7) | *AG* (47.1) | *AA* (34.2) |  | 0.34 |
| rs749256 | 156884365 | 15318 | intron | 0.009 |  | *T* (22.8) | *C* (77.2) |  | *TT* (6.7) | *CT* (32.2) | *CC* (61.1) |  | 0.01 |

*A1* and *A2* refer to the minor and major alleles; *A1A1*, *A2A1* and *A2A2* indicate homozygotes and heterozygotes of the minor allele and homozygotes of the major allele, respectively. Base pairs and *r2* are measures for the distance from and the linkage disequilibrium with rs12566888. *P*-values are for departure from Hardy–Weinberg equilibrium.

**Table S3**

Allele and genotype frequencies in all 1938 participants

| SNPs |  | Base pairs |  | Allele frequencies | |  | Genotype frequencies | | |
| --- | --- | --- | --- | --- | --- | --- | --- | --- | --- |
|  |  | *A1* / *A2* |  | *A1* | *A2* |  | *A1A1* | *A2A1* | *A2A2* |
| rs2768762 |  | *G / T* |  | 819 (21.1) | 3057 (78.9) |  | 92 (4.7) | 635 (32.8) | 1211 (62.5) |
| rs2644620 |  | *C / T* |  | 473 (12.2) | 3403 (87.8) |  | 32 (1.7) | 409 (21.1) | 1497 (77.2) |
| rs12566888 |  | *T / G* |  | 385 (9.9) | 3491 (90.1) |  | 22 (1.1) | 341 (17.6) | 1575 (81.3) |
| rs2768744 |  | *G / A* |  | 822 (21.2) | 3054 (78.8) |  | 82 (4.2) | 658 (34.0) | 1198 (61.8) |
| rs6671392 |  | *C / T* |  | 531 (13.7) | 3345 (86.3) |  | 41 (2.1) | 449 (34.0) | 1448 (61.8) |
| rs822441 |  | *C / G* |  | 625 (16.1) | 3251 (83.9) |  | 57 (2.9) | 511 (26.4) | 1370 (70.7) |
| rs11264581 |  | *A / G* |  | 623 (16.1) | 3253 (83.9) |  | 47 (2.4) | 529 (27.3) | 1362 (70.3) |
| rs12137505 |  | *G / A* |  | 1663 (42.9) | 2213 (57.1) |  | 359 (18.5) | 945 (48.8) | 634 (32.7) |
| rs749256 |  | *T* / *C* |  | 860 (22.2) | 3016 (77.8) |  | 105 (5.4) | 650 (33.5) | 1183 (61.0) |

*A1* and *A2* refer to the minor and major alleles; *A1A1*, *A2A1* and *A2A2* indicate homozygotes and heterozygotes of the minor allele and homozygotes of the major allele, respectively. Values are number of alleles or genotypes (%).

**Table S4**

Multivariable-adjusted hazard ratios for the all-cause mortality and the composite cardiovascular endpoint by *PEAR1* SNPs

| SNP |  | N° of events / N° at risk (%) | |  | Hazard ratio  (95% CI) |  | *P* |
| --- | --- | --- | --- | --- | --- | --- | --- |
|  |  | Minor allele  carriers | Major allele homozygotes |  |  |  |  |
| Total mortality |  |  |  |  |  |  |  |
| *rs2768762* |  | *[G]* 90/727 (12.4) | *[TT]* 148/1211 (12.2) |  | 1.03 (0.80–1.33) |  | 0.82 |
| *rs2644620* |  | *[C]* 57/441 (12.9) | *[TT]* 181/1497 (12.1) |  | 0.99 (0.74–1.32) |  | 0.92 |
| rs12566888 |  | *[T]* 40/363 (11.0) | *[GG]* 198/1575 (12.6) |  | 0.99 (0.72–1.36) |  | 0.95 |
| rs2768744 |  | *[G]* 88/740 (11.9) | *[AA]* 150/1198 (12.5) |  | 1.01 (0.76–1.33) |  | 0.96 |
| rs6671392 |  | *[C]* 58/490 (11.8) | *[TT]* 180/1448 (12.4) |  | 0.95 (0.72–1.26) |  | 0.73 |
| rs822441 |  | *[C]* 72/568 (12.7) | *[GG]* 166/1370 (12.1) |  | 1.07 (0.84–1.37) |  | 0.56 |
| rs11264581 |  | *[A]* 75/576 (13.0) | *[GG]* 163/1362 (12.0) |  | 1.06 (0.81–1.40) |  | 0.67 |
| rs12137505 |  | *[G]* 163/1304 (12.5) | *[AA]* 75/634 (11.8) |  | 0.87 (0.65–1.16) |  | 0.35 |
| Cardiovascular events |  |  |  |  |  |  |  |
| *rs2768762* |  | *[G]* 70/727 (9.6) | *[TT]* 111/1211 (9.2) |  | 1.13 (0.83–1.52) |  | 0.44 |
| *rs2644620* |  | *[C]* 48/441 (10.9) | *[TT]* 133/1497 (8.9) |  | 1.28 (0.91–1.79) |  | 0.15 |
| rs12566888 |  | *[T]* 27/363 (7.4) | *[GG]* 154/1575 (9.8) |  | 0.78 (0.50–1.20) |  | 0.25 |
| rs2768744 |  | *[G]* 68/740 (9.2) | *[AA]* 113/1198 (9.4) |  | 1.00 (0.74–1.35) |  | 0.98 |
| rs6671392 |  | *[C]* 48/490 (9.8) | *[TT]* 133/1448 (9.2) |  | 1.14 (0.79–1.64) |  | 0.50 |
| rs822441 |  | *[C]* 63/568 (11.1) | *[GG]* 118/1370 (8.6) |  | 1.30 (0.94–1.80) |  | 0.11 |
| rs11264581 |  | *[A]* 54/576 (9.4) | *[GG]* 127/1362 (9.3) |  | 1.02 (0.70–1.47) |  | 0.93 |
| rs12137505 |  | *[G]* 122/1304 (9.4) | *[AA]* 59/634 (9.3) |  | 0.94 (0.67–1.33) |  | 0.73 |

Hazard ratios (95% confidence interval) express the risk of minor allele carriers *vs.* major allele homozygotes. Hazard ratios account for family clusters, and were adjusted for baseline characteristics including sex, age, body mass index, mean arterial pressure, total-to-HDL cholesterol ratio, smoking and drinking, antihypertensive drug treatment, and history of cardiovascular disease and diabetes mellitus.

**Table S5**

Multivariable-adjusted hazard ratios associated with *rs12566888* by antiplatelet treatment status

| Event | |  | | On antiplatelet drugs | | | | | |  | | | Not on antiplatelet drugs | | | | | | |  | | |  | | |
| --- | --- | --- | --- | --- | --- | --- | --- | --- | --- | --- | --- | --- | --- | --- | --- | --- | --- | --- | --- | --- | --- | --- | --- | --- | --- |
|  |  |  | | *T* allele carriers *vs.* *GG* homozygotes | | | | | | |  | | | *T* allele carriers *vs.* *GG* homozygotes | | | | | | |  | | |  | |
|  |  |  | | N° events | | Hazard ratio | | *P* | | |  | | | N° events | | Hazard ratio | | *P* | | |  | | | *P*int | |
| N° at risk |  | | 50 *vs.* 200 | | | | | |  | | | 313 *vs.* 1735 | | | | | | |  | | |  | | |  |
| Total mortality |  | | 9 *vs*.38 | | 0.71 (0.32–1.60) | | 0.40 | |  | | | 31 *vs.* 160 | | | 0.98 (0.69–1.40) | | 0.91 | |  | | | 0.65 | | |  |
| Cardiovascular mortality |  | | 2 *vs*. 13 | | 0.66 (0.14–3.12) | | 0.60 | |  | | | 5 *vs*. 33 | | | 0.87 (0.35–2.15) | | 0.76 | |  | | | 0.51 | | |  |
| Cardiovascular events |  | | 10 *vs*. 45 | | 0.93 (0.47–1.83) | | 0.83 | |  | | | 17 *vs*. 109 | | | 0.72 (0.41–1.26) | | 0.24 | |  | | | 0.75 | | |  |
| Coronary events |  | | 6 *vs*. 30 | | 0.94 (0.42–2.07) | | 0.87 | |  | | | 12 *vs*. 59 | | | 0.94 (0.48–1.85) | | 0.86 | |  | | | 0.83 | | |  |
| Ischaemic cerebrovascular events |  | | 4 *vs*. 13 | | 1.07 (0.34–3.44) | | 0.90 | |  | | | 4 *vs*. 40 | | | 0.52 (0.18–1.49) | | 0.20 | |  | | | 0.30 | | |  |

Numbers of events do not add up, because only the first event in each category was analysed. Hazard ratios (95% confidence interval) express the risk of minor allele carriers *vs.* major allele homozygotes, account for family clusters, and were adjusted for baseline characteristics including sex, age, body mass index, mean arterial pressure, total-to-HDL cholesterol ratio, smoking and drinking, antihypertensive drug treatment and history of cardiovascular disease and diabetes mellitus. *P* and *P*int indicate the significance of the hazard ratios within subgroups and of the genotype-by-treatment-status interaction.

**Table S6**

**Frequency of reconstructed haplotypes**

| Haplotype | N° (%) |  | Haplotype | N° (%) |
| --- | --- | --- | --- | --- |
| *TTGATGGA* | 1438 (37.1) |  | *GTTGTCAG* | 3 (0.077) |
| *TTGATGGG* | 883 (22.8) |  | *TTGACGAG* | 3 (0.077) |
| *TTGATGAG* | 465 (12.0) |  | *TTTATGGG* | 3 (0.077) |
| *GCGGCCGA* | 266 (6.86) |  | *GCTGCCGA* | 2 (0.052) |
| *GTTGTGAG* | 140 (3.61) |  | *GCTGTCGG* | 2 (0.052) |
| *GTTGTCGA* | 109 (2.81) |  | *TCGGCCGA* | 2 (0.052) |
| *TTGACCGA* | 97 (2.50) |  | *TTGACGGG* | 2 (0.052) |
| *GCGGCGGG* | 83 (2.14) |  | *TTGGCGGA* | 2 (0.052) |
| *TTGATCGA* | 53 (1.37) |  | *TTGGTGGA* | 2 (0.052) |
| *GCGGTGGA* | 40 (1.03) |  | *GCGACCAG* | 1 (0.026) |
| *GTTGTGGA* | 36 (0.93) |  | *GCGATGAG* | 1 (0.026) |
| *TTGGCCGA* | 36 (0.93) |  | *GTGGTCGA* | 1 (0.026) |
| *GTGATGGA* | 35 (0.90) |  | *GTGGTGAG* | 1 (0.026) |
| *TTTGTGGG* | 32 (0.83) |  | *GTTGCCAG* | 1 (0.026) |
| *TTTGTCGA* | 27 (0.70) |  | *GTTGTGAA* | 1 (0.026) |
| *GCGATGGG* | 24 (0.62) |  | *GTTGTGGG* | 1 (0.026) |
| *GTTATGGA* | 19 (0.49) |  | *TCGACCAG* | 1 (0.026) |
| *GCGGCGGA* | 16 (0.41) |  | *TCGGCGGG* | 1 (0.026) |
| *GCGATGGA* | 15 (0.39) |  | *TTGACGGA* | 1 (0.026) |
| *GCGGCCGG* | 9 (0.23) |  | *TTGATGAA* | 1 (0.026) |
| *GCGGTCGA* | 6 (0.15) |  | *TTGGCCGG* | 1 (0.026) |
| *GCGACCGA* | 4 (0.10) |  | *TTGGTCGG* | 1 (0.026) |
| *TTTATGAG* | 4 (0.10) |  | *TTTATGGA* | 3 (0.077) |
| *GTTACCGA* | 3 (0.077) |  | *TTTGTGAG* | 1 (0.026) |

From top to bottom and from left to right, haplotypes are order by frequency (%). Using the expectation-maximisation algorithm as implemented in the PROC HAPLOTYPE procedure of the SAS software, we used all SNPs included in the statistical analysis to reconstruct haplotypes.

**Table S7**

Multivariable-adjusted hazard ratios associated with the *TTGATGGA* haplotype by antiplatelet treatment status

| Event |  | On antiplatelet drugs | | |  | Not on antiplatelet drugs | | |  |  |
| --- | --- | --- | --- | --- | --- | --- | --- | --- | --- | --- |
|  |  | *TTGATGGA* carriers *vs.* non-carriers | | |  | *TTGATGGA* carriers *vs.* non-carriers | | |  |  |
|  |  | N° events | Hazard ratio | *P* |  | N° events | Hazard ratio | *P* |  | *P*int |
| N° at risk |  | 140 *vs.* 110 | | |  | 1034 *vs.* 654 | | |  |  |
| Total mortality |  | 27 *vs*. 20 | 1.46 (0.76–2.80) | 0.26 |  | 119 *vs.* 72 | 1.03 (0.77–1.36) | 0.86 |  | 0.57 |
| Cardiovascular mortality |  | 10 *vs*. 5 | 1.84 (0.57–6.00) | 0.31 |  | 19 *vs*. 19 | 0.59 (0.30–1.15) | 0.12 |  | 0.39 |
| Cardiovascular events |  | 34 *vs*. 21 | 1.38 (0.77–2.47) | 0.28 |  | 68 *vs*. 58 | 0.69 (0.47–1.02) | 0.059 |  | 0.056 |
| Coronary events |  | 23 *vs*. 13 | 1.43 (0.73–2.82) | 0.29 |  | 41 *vs*. 30 | 0.78 (0.46–1.33) | 0.37 |  | 0.078 |
| Ischaemic cerebrovascular events |  | 8 *vs*. 9 | 0.75 (0.25–2.25) | 0.61 |  | 23 *vs*. 21 | 0.64 (0.36–1.13) | 0.12 |  | 0.72 |

Numbers of events do not add up, because only the first event in each category was analysed. Hazard ratios (95% confidence interval) express the risk of minor allele carriers *vs.* major allele homozygotes, account for family clusters, and were adjusted for baseline characteristics including sex, age, body mass index, mean arterial pressure, total-to-HDL cholesterol ratio, smoking and drinking, antihypertensive drug treatment and history of cardiovascular disease and diabetes mellitus. *P* and *P*int indicate the significance of the hazard ratios within subgroups and of the genotype-by-treatment-status interaction. Three haplotypes with a frequency of over 10% were tested. To retain significance while accounting for multiple testing, *P*‑values should be less than 0.017.

**Table S8**

Minor allele frequencies in current study population and in European subjects in *1000 Genomes* and *HapMap*

| **SNP** |  | ***Minor allele*** |  | ***Current study*** | |  | ***1000 Genomes*** | |  | ***HapMap*** | |
| --- | --- | --- | --- | --- | --- | --- | --- | --- | --- | --- | --- |
|  |  |  |  | **N** | **Frequency** |  | **N** | **Frequency** |  | **N** | **Frequency** |
| rs2768762 |  | *G* |  | 787 | 21.5 |  | 503 | 22.0 |  | 346 | 15.9 |
| rs2644620 |  | *C* |  | 787 | 12.5 |  | 503 | 12.2 |  | 116 | 11.2 |
| rs12566888 |  | *T* |  | 787 | 10.4 |  | 503 | 9.24 |  | 216 | 4.63 |
| rs2768744 |  | *G* |  | 787 | 22.0 |  | 503 | 20.6 |  | 112 | 16.1 |
| rs6671392 |  | *C* |  | 787 | 14.0 |  | 503 | 12.4 |  | 226 | 10.6 |
| rs822441 |  | *C* |  | 787 | 16.1 |  | 503 | 15.9 |  | 238 | 12.6 |
| rs11264581 |  | *A* |  | 787 | 16.6 |  | 503 | 17.6 |  | 226 | 13.3 |
| rs12137505 |  | *G* |  | 787 | 42.2 |  | 503 | 39.3 |  | 344 | 35.5 |
| rs749256 |  | *T* |  | 787 | 22.8 |  | 503 | 26.8 |  | 226 | 23.9 |

N  refers to the number of subjects included in the frequency calculations, restricted to White Europeans included in the *1000 Genomes* (https://www.ncbi.nlm.nih.gov/variation/tools/1000genomes/) and *HapMap* (ftp://ftp.ncbi.nlm.nih.gov/hapmap/) databases.
